# Notes and News

**Published:** 1890-02-22

**Authors:** 


### Notes and News.

Collected and Communicated.

The Hospitals Association holds the fifth meeting of this session, on Wednesday, the 26th February, in the Board Room of Charing Cross Hospital. The chair will be taken by Mr. E. Murray Ind, chairman of the London Hospital, at half-past eight p.m. A paper will be read by Mr. Burdett, on "The Financial Aspects of the Hospital Problem, and How to Remedy some existing Defects." This paper will be illustrated by diagrams, and a complete system of book-keeping and reports will be explained. Tickets of admission may be obtained on application to the secretary, at Norfolk House, Norfolk Street, Strand.

Worcester has been very lively lately; it has started the Worcestershire Health Society, and a branch of the Society for the Prevention of Cruelty to Children. At the County Council meeting also it was proposed to appoint a Medical Officer of Health at a salary of £800 a-year, and the proposal has called forth much discussion. The local Convalescent Home for Women has also held its annual gathering, and was able to report twenty-six cases assisted, and a balance in hand of £39.

Dorset County Hospital issues an annual report, which says that the total number of in-patients treated during the year had decreased from 352 in 1888, to 341, and the weekly average had fallen from 41 to 39. The out-patients had increased from 786 to 874, and the casual patients from 728 to 768. The income of the year arising from subscriptions exceeded that of 1888 by £12 17s.; from collections by £4 10s. 1d.; from ordinary donations by £61 9s.; and from invested capital by £9 2s. 9d. The expenditure was less by £54 0s. 7d.

At the annual meeting of the South Devon and East Cornwall Hospital, on February 7th, the Earl of Morley took the chair, but immediately said he wished to vacate it for this reason, that fifty years ago, when the first annual meeting of the hospital was held, Sir Ralph Lopes was in the chair. It would, he thought, be gratifying to Sir Massey Lopes and to themselves to have his name as presiding at their fiftieth annual meeting—the jubilee meeting of the hospital—when they remembered how deep a debt of gratitude the hospital owed to him in many ways. He would, therefore, ask Sir Massey Lopes to take the chair on that occasion. The report submitted was satisfactory.

The Ashburton and Buckfastleigh Cottage Hospital continues to work satisfactorily. The receipts last year were £206 10s., the expenditure £206 17s., the expenses of opening the new hospital having been £6. Over forty cases were treated last year; there were six deaths. Miss Elliott, who trained at St. Thomas's, is matron-nurse.

At the annual meeting of subscribers to Rotherham Hospital it was stated that 269 in-patients and 5,116 out-patients were treated last year. Mr. J. Gibbs read the annual financial statement. The subscriptions from individuals and firms had amounted to £581 1s.; ditto from workmen, £774 5s. 10d.; collections, churches and chapels, £246 12s. 3d. The invested funds amounted to £6,790 9s. 6d. We are glad to see Miss Hale is still matron.

Leprosy has broken out among the natives belonging to the tribe of Momea, New Caledonia, numbers of whom are tainted with it; and a free colonist of Moindon, who has been in the habit of associating with them, has also been attacked by the loathsome malady. Steps are being taken for the isolation of the sufferers. A lazar-house has been established on an island in the Bay of Dumbea, and another at Pic des Morts, in the district of Canala.

The West Cornwall Women's Hospital at Redruth has been formally opened by its President, Mrs. Basset. A large number of people were present, especially ladies, and Mr. Tweedy told the story of the movement which had resulted in the erection of the Hospital. In the winter of 1886 several medical gentlemen and others pointed out to Mr. and Mrs. Basset that a hospital for the women employed in the mines of the neighbourhood would be a great boon. Mr. and Mrs. Basset took the subject up warmly, and Mr. Basset retained his interest in it until his untimely death. The scheme was heartily taken up and carried through triumphantly, and Redruth is to be congratulated on its pretty new hospital.

The annual meeting of the Royal Free Hospital was held last week. The financial statement showed that the total income for the year had been £9,587 3s. 11d., as compared with £12,327 9s. in 1888, the ordinary receipts being £4,583 against £6,761, and the legacies £4,993, as compared with £5,565. The ordinary expenditure amounted to £9,965 19s. 7d., as compared with £10,336 2s. 7d. in 1888, and £10,890 12s. 7d. in 1887. The medical reports showed that the resources of the hospital had been fully utilized, the number of in-patients admitted being 2,005, as compared with 1,823 in 1888. The number of out-patients had also shown an increase on the previous year. The medical school contains 102 students, and very gratifying successes had been won by them. An earnest hope is expressed that money will shortly be forthcoming to rebuild the front of the hospital.

The Coventry centre of the St. John's Ambulance Association was honoured by the presence of Sir H. C. Perrott at the late distribution of certificates. At this annual meeting there were 35 to receive certificates, 10 second certificates, and 9 medallions. The lecturer for the first seven sessions was the late Dr. Arthur Read, since whose decease Dr. Haig, his successor, had taken the classes. In the women's classes 915 had attended the first aid and nursing, 247 had passed, and 32 had obtained medallions. A class had also been held at Stoke, at which there had been 45 attending, of whom 10 were to receive certificates. An ambulance brigade had been formed amongst the certificate holders, and about 30 had joined the brigade meeting. Dr. Milner Moore had very kindly presented to the brigade a complete transport stretcher, and sets of splints, tourniquets, and bandages had been purchased. The mayor, Sir H. C. Perrott, and Mr. Gulson all spoke well in favour of the association.

The following vacancies are announced this week, the amount of the salary in each case being given after the name of the institution:

**Resident Medical Officers.**

City Road Hospital for Diseases of the Chest. Medical Officer—£100, board, etc.
National Hospital, Queen Square. Senior House Physician—£100, board, etc.
National Hospital, Queen Square. Junior House Physician—£50, board, etc.
National Dental Hospital. House Surgeon.
Wolverhampton Eye Infirmary. Resident Assistant—Board, etc.
Guildford County Hospital. House Surgeon—£80, board, etc.
North Staffordshire Infirmary. House Physician—£100, board, etc.
Liverpool Northern Hospital. House Physician—£80, board, etc.
Newcastle City Hospital. Medical Assistant—£50, board, etc.
Birmingham Homœopathic Hospital, House Surgeon—£80, Board, etc.
Boyle Union, Medical Officer for Workhouse—£100.
Boyle Union, Medical Officer for Dispensary—£95.
Gainsborough Friendly Societies, Medical Officer—£160.
Banstead Asylum, Senior Assistant Medical Officer—£250.
Banstead Asylum, Fourth Assistant Medical Officer—£100.
Banstead Asylum, Dispenser—£100.

**Non-Resident Medical Officers.**

Highworth Union, Medical Officer—£70.

Hampstead Provident Dispensary, Medical Officer.

Finsbury Dispensary Surgeon—£40.

Burton-on-Trent Medical Association, Junior Assistant.

National Dental Hospital, Assistant Surgeon.

Farringdon Dispensary, Honorary Surgeon.

Victoria Park Chest Hospital, Association Physician.

---

So Mr. Howie-Boyd has been beaten at Cumberland Infirmary, and instead of more medical officers to attend to the out-patients, there is to be a Dispenser appointed at £25 a-year. We hope the committee will find this plan answer, but we doubt it. They would have done better to follow the advice of their senior physician.

---

Last Saturday there was a meeting of the delegates of the Hospital Saturday Fund. Mr. R. B. D. Acland presided, and the following resolutions were unanimously passed: "That the Hospital Saturday Fund being incorporated under the Companies Acts, all the interest in any property now belonging to or held in trust for the board of delegates of the Hospital Saturday Fund be transferred to the association so incorporated, and that the business of the fund be conducted in accordance with the provisions of the articles of association," and "That the existing governing body be and is hereby dissolved." The secretary, Mr. R. Frewer, announced that subscriptions to the amount of £1,681 5s. 5d. had been received up to Friday night on account for the current year. The first meeting of the fund under the new incorporation will take place to-day at the offices.

---

On Friday, February 8th, Mr. John Godson, at the Jaffray Hospital, entertained at a "farewell tea" the resident medical and nursing staff, the occasion commemorating the termination of his term of office as resident assistant house surgeon for three months. The invitations were issued the day before for tea at half-past three, the facetious information being added that Bath chairs and the lift would be available for use at five p.m. The company included Dr. Ballance (house surgeon), Miss Wemyss (matron), Miss Williams (General Hospital), Mr. Hawley (present assistant), and Mr. Warneford (a visitor to the Jaffray). The other guests consisted of nearly all the nurses of the establishment. Due justice having been done to the tea and coffee, and bread and butter, cakes and biscuits, speeches became the order of the evening, the toasts being proposed in tea, a method of procedure that no doubt would be very delightful to Sir Wilfrid Lawson's teetotal proclivities. "The Health of the Host," Mr. Godson, was proposed by the Matron, and seconded in flattering terms by Dr. Ballance.

The Princess Alice Memorial Hospital is greedy; at the suggestion of the Rev. H. C. Wilson it is going to have two Hospital Sundays in each year. Still the hospital has some excuse, for it has treated over forty more patients this year than last, and has its splendid new buildings to fill and to maintain.

---

Hyderabad is just now the cynosure of all eyes, and Hyderabad is determined to be worthy of the notice it has excited in medical circles. A large new hospital, to hold 300 beds, is being erected, and orders are being received in London for all the latest and best appliances and fittings. The beds are to be Howe's patent spring bedsteads, which are cheap, cleanly, and easy to pack for long journeys. It is strange that more of our English hospitals have not adopted these clever Yankee beds, for their low price and convenient make eminently fit them for use in large institutions. The St. Anne's Convalescent Home and the Royal Orthopædic have them, but they are not so widely known as they deserve to be.

---

There can be no doubt that the Whitehall Rooms have the best accommodation for an afternoon meeting on behalf of a charity. Centrally situated, easily accessible, lighted by electricity, and of sufficient size, the room in question is unequalled for such a purpose. These facts were proved to demonstration at the annual court of the Dreadnought Seamen's Hospital on Tuesday, under the presidency of Lord George Hamilton, the First Lord of the Admiralty. There was a crowded and enthusiastic audience, comprising some of the oldest and best known of our admirals. Amongst the speakers were three octogenarians, one speaker being 92 years old. This large and representative gathering assembled to do honour to Mr. F. Cleeve, C.B., for eighteen years a member of the Committee of Management, during twelve of which he acted as deputy-chairman and three as chairman. Mr. Cleeve was presented with his portrait, an excellent likeness and a work of art, painted by Mr. Arthur Hacker, who has already attained fame as a portrait painter. No one has done more for the British sailor than Mr. Cleeve, and it is well that his life's work should be thus fittingly commemorated in perpetuity. Lord George was in his happiest vein, and made a really able speech. Everybody was pleased, and the old Dreadnought ought to largely benefit by the meeting.

---

The Victoria Infirmary, Glasgow, was opened on Friday, 14th inst., by his Grace the Duke of Argyle. It is on the south side of the city, where many of the largest public works are situated; and stands upon the exact spot where the fiercest action in the battle of Langside took place. His Grace the Duke of Argyle, the Lord Provost, and magistrates were received by the directors and managers, and after visiting part of the infirmary, met in the large ward for refreshments. The ladies, accompanied by the matron, went over the principal wards before having tea, which was served in one of the smaller rooms. After this the company proceeded to Langside Established Church, which was already crowded. After the singing of Psalm cxxi., Scripture reading, and prayer, an historical account of the origin of the institution was given. His Grace the Duke of Argyle then gave an address, touching upon the work of hospitals, and upon the extreme need of this new infirmary in Glasgow. The services closed with a hymn and prayer, when those present in church inspected the infirmary. The wards are extremely pretty, painted light green, with a dado of cream-coloured tiles, and with floors of light polished wood. The matron, Miss Ross, is a most attractive woman, and the visitors were all greatly pleased with their tour of inspection under her guidance. There are about 80 beds, six of which are for private patients. There is not nearly so much corridor space as in the Western.

